# The Actual State of Sexual Dysfunction in Newlywed Men Starting to Try for a Baby in Japan

**DOI:** 10.1002/rmb2.70023

**Published:** 2026-01-28

**Authors:** Ayumu Taniguchi, Akimasa Kure, Yuki Nagashima, Ippei Hiramatsu, Masato Shirai, Kazuhiro Kobayashi, Akira Tsujimura

**Affiliations:** ^1^ Department of Urology Juntendo University Urayasu Hospital Chiba Japan; ^2^ Department of Urology Juntendo University Shizuoka Hospital Shizuoka Japan; ^3^ Department of Urology Graduate School of Medicine, Juntendo University Tokyo Japan; ^4^ D Clinic Tokyo Tokyo Japan

**Keywords:** age, depression, obesity, sexual dysfunction

## Abstract

**Purpose:**

We aimed to evaluate the sexual function in newlywed men or men just before marriage to better understand the current situation of the male sexual and reproductive function.

**Methods:**

This study included 719 men visiting our hospital or clinic for fertility screening. Patient characteristics including age, sexual status, semen parameters, several symptoms, and blood analysis results were assessed. We compared several factors between patients with sexual dysfunction (SD group) and those with normal sexual function (SN group), and performed univariate and multivariate analyses to identify independent risk factors for sexual dysfunction.

**Results:**

Of 719 men, 139 (19.3%) were identified as having sexual dysfunction, including erectile dysfunction [ED], *n* = 88 [12.2%]; ejaculatory dysfunction, *n* = 66 [9.1%]; and decreased libido, *n* = 35 [4.9%]. Significant differences between the SD and SN groups were observed in age, albumin, liver enzymes, triglycerides, blood sugar, and semen volume. In a multivariate analysis, only age, body mass index, and the Beck Depression Inventory score for depression remained significant risk factors for sexual dysfunction.

**Conclusions:**

It was considered that men who are older, obese, or exhibit depressive symptoms are likely to experience sexual dysfunction before the initiation of fertility treatment.

## Graphical abstract

We found that 139 (19.3%) in 719 newlywed men or men just before marriage were identified as having sexual dysfunction at our bridal examination. It was considered that men who are older, obese, or exhibit depressive symptoms are likely to experience sexual dysfunction before the initiation of fertility treatment.

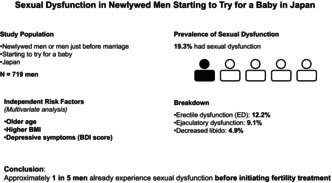

## 1 Introduction

The number of births in Japan has been declining annually, falling to 686 061 in 2024, and below 700 000 for the first time. The total fertility rate also dropped to a historic low of 1.15. Japan is in the midst of a national crisis caused by its declining birthrate. According to a survey conducted by the Ministry of Health, Labour and Welfare, as of 2015, approximately 1 in 5.5 couples in Japan had undergone infertility testing or treatment. By 2021, this ratio had increased to 1 in 4.4 couples. Thus, addressing infertility is extremely important as a solution to the declining birthrate. Furthermore, recent studies have reported a progressive decline in semen quality over time, with an accelerating decrease in sperm count [1, 2]. Although the precise etiology remains unclear, it is hypothesized that modern lifestyle factors such as obesity, sleep deprivation, and physical inactivity may be contributing to this trend. Notably, while the WHO previously reported in 1993 that male factors were responsible in 48% of infertile couples, it is speculated that this proportion may be even higher today due to the deterioration of semen quality over time. A 2015 nationwide survey on the examination and treatment of male infertility in Japan found that impaired spermatogenesis accounted for more than 80% of male infertility cases [3]. In our previous study involving 564 men (mean age, 35.4 ± 6.9 years) who were beginning attempts to conceive, approximately one‐fourth already failed to meet the World Health Organization (WHO) reference values for semen quality [4]. This ratio is also extremely noteworthy, but what is even more interesting is that the prevalence of sexual dysfunction as a cause of male infertility has increased significantly, from 3.3% in a 1997 nationwide survey to 13.5% in 2015 [3]. This trend is further supported by the 2023 national survey on male sexual dysfunction conducted by the Japanese Society for Sexual Medicine (JSSM), which revealed a notable decline in sexual function among young men [5, 6, 7]. Specifically, a high prevalence of erectile dysfunction (ED; defined as the inability to achieve penetration during intercourse) and decreased libido was observed among young men. In fact, the prevalence of ED in males aged 20–24, 25–29, and 30–34 years was 26.6%, 22.0%, and 16.4%, respectively. Furthermore, the rate of those who reported having no or little sexual desire in males aged 20–24, 25–29, and 30–34 years was 21.2%, 14.7%, and 15.8%, respectively.

Despite these findings, data on the prevalence of sexual dysfunction among newlywed men or men approaching marriage remain limited. Therefore, we aimed to evaluate the sexual function in this specific population of men who are presumed to be at their peak of sexual activity, in order to better understand the current state of male sexual health in the context of fertility.

## 2 Patients and Methods

### 2.1 Patients

This study included 719 newlywed men or men just before marriage who underwent various investigations in our hospital or affiliated clinics between October 2014 and June 2018, hoping for the examination of sexual function and semen findings. Patients who had previously undergone a semen analysis and were referred to our facility due to any abnormality were excluded. Approximately half were just before marriage (within 3 months), while the remaining half were within 1 year in principle after marriage, just before starting fertility efforts. They were considered to be a generally healthy and standard group of men. Patient characteristics including age, sexual status, semen parameters, scores of symptom‐specific questionnaires, and blood analysis results were assessed.

### 2.2 Methods

We directly interviewed patients undergoing their first semen analysis and assessed their sexual function. The sexual dysfunction was determined by a simple dichotomous determination of whether the patient feels that they have a disorder. Based on their reports, we categorized those with ED, ejaculatory dysfunction, decreased libido, or multiple symptoms as having sexual dysfunction. We compared age, body mass index (BMI), smoking habit, testicular volume, semen parameters (including semen volume, sperm count, and sperm motility), the scores of symptom‐specific questionnaires (the International Prostate Symptom Score [IPSS] for lower urinary tract symptoms, the Beck Depression Inventory [BDI] for depression, and the Sexual Health Inventory for Men [SHIM] and Erection Hardness Score [EHS] for erectile function), which were routinely obtained, and several biochemical and endocrinological factors including luteinizing hormone, follicle‐stimulating hormone, and total testosterone between patients with sexual dysfunction (SD group) and those with normal sexual function (SN group). All blood samples were collected between 09:00 and 11:00 to monitor endocrinological variables. A semen test was performed after ≥ 4 days of abstinence by use of a Makler semen counting chamber. The semen analysis was basically performed in accordance with the WHO Laboratory Manual. Subsequently, to identify independent factors associated with sexual dysfunction, we performed a univariate analysis using factors after selecting representative factors that could be confounding variables. Finally, a multivariate analysis was performed that included factors identified as significant in the univariate analyses. This study was approved by the institutional review board of Juntendo University Urayasu Hospital (approval no. 2018‐033).

### 2.3 Data Analysis

The Man‐Whitney *U* test was used to compare several factors between the SD and SN groups. Statistical significance was set at *p* < 0.05. Further, factors predicting sexual dysfunction were identified using a logistic univariate analysis. In univariate analysis, age was categorized into four groups, with the group aged 21 years or older but under 30 years serving as the reference. Similarly, BMI was categorized into four groups, with the group with a BMI between 20.8 and 22.5 (closer to standard body type) serving as the reference for analysis. For smoking history, the group with no smoking history was used as the reference. Testicular volume was categorized into four groups, with the largest group as the reference. IPSS and BDI were also categorized into four groups, with the group having the lowest scores used as the reference for analysis. Blood test results, as shown in Table 4, were categorized into four groups for each item, with the group considered clinically favorable used as the reference for analysis. Factors with *p* < 0.05 in the univariate analyses were incorporated into the multivariate analysis model. Then, the logistic multivariate analysis, using backward elimination, was conducted to identify independent predictive factors (*p* < 0.05). Statistical analyses were conducted using SPSS (ver. 24.0, SPSS, Chicago, IL, USA).

## 3 Results

Patient characteristics are shown in Table 1. All measurements are presented as mean ± standard deviation. Patient age and BMI were 35.5 ± 6.5 years and 22.8 ± 3.0 kg/m^2^. One hundred thirteen men (15.7%) were smokers. The testicular volume of both testicles was almost 20 mL. The IPSS (3.4 ± 3.7) and BDI (5.4 ± 5.4) scores were determined to be within normal ranges, while the SHIM score (18.9 ± 5.2) fell within the mild ED category. However, EHS (3.5 ± 0.7) scores exceeded 3 points, indicating this is not a population where the erectile function itself is significantly diminished. The mean values of biochemical tests, endocrine tests, and semen analysis findings all remained within the normal ranges. Table 2 shows the symptoms of sexual dysfunction. Of 719 men in the present study, 139 (19.3%) were identified as having sexual dysfunction. While some overlap in symptoms existed, 88 individuals (12.2%) reported ED, 66 (9.1%) reported ejaculatory dysfunction, and 35 (4.9%) reported decreased libido. Table 3 shows the comparison between the SD and SN groups. The mean age of the SD group (38.4 ± 7.6 years) was significantly higher than the SN group (34.8 ± 6.1 years; *p* < 0.01). SHIM and EHS scores were significantly higher in the SN group, as expected, while IPSS and BDI were significantly lower in the SN group. Significant differences were observed between the two groups in albumin, liver enzymes, triglycerides, and FBS. No significant differences were found in endocrine factors. The results of the logistic analysis are shown in Table 4. Univariate analyses identified the following 12 associated factors: age, BMI, history of smoking, testicular volume, IPSS and BDI scores, total protein, ALT, creatinine, triglyceride, HbA1c, and testosterone. SHIM and EHS were excluded as they are directly related to sexual function. Representative factors were selected from those suspected to be confounders. In the multivariate analysis of the six selected factors that showed statistical significance in the univariate analyses, only age, BMI, and BDI score retained their significant association with sexual dysfunction. As a result, it was considered that men who are older, tend to be obese, or exhibit depressive symptoms likely experience sexual dysfunction even before beginning fertility treatment.

**TABLE 1:** Clinical characteristics of 719 men just before wedding or newly wedded.

*N*	719
Age (years)	35.5 ± 6.5
BMI (kg/m^2^)	22.8 ± 3.0
Smoking (%)	15.7
Rt. testicular volume (mL)	19.6 ± 4.3
Lt. testicular volume (mL)	20.7 ± 3.9
IPSS	3.4 ± 3.7
BDI	5.4 ± 5.4
SHIM	18.9 ± 5.2
EHS	3.5 ± 0.7
TP (g/dL)	7.4 ± 0.4
Albumin (g/dL)	4.8 ± 0.3
AST (U/L)	22.9 ± 12.8
ALT (U/L)	26.7 ± 23.5
γ‐GTP (U/I)	36.5 ± 35.8
BUN (mg/dL)	13.0 ± 2.9
Creatinine (mg/dL)	0.8 ± 0.1
T‐cholesterol (mg/dL)	196.3 ± 33.0
Triglyceride (mg/dL)	95.1 ± 68.5
LDL‐cholesterol (mg/dL)	120.0 ± 30.6
HDL‐cholesterol (mg/dL)	60.0 ± 13.3
FBS (mg/dL)	87.1 ± 9.4
HbA1c (%)	5.3 ± 0.3
LH (IU/L)	3.4 ± 1.8
FSH (IU/L)	4.3 ± 3.8
Total testosterone (ng/mL)	5.4 ± 1.8
Semen volume (mL)	3.3 ± 1.6
Sperm count (×10^6^/mL)	70.5 ± 42.3
Sperm motility (%)	62.1 ± 17.1

*Note:* All measurements are presented as mean ± standard deviation.

Abbreviations: γ‐GTP, gamma‐glutamyl transpeptidase; ALT, alanine aminotransferase; AST, aspartate aminotransferase; BDI, Beck Depression Inventory; BMI, body mass index; BUN, blood urea nitrogen; EHS, Erection Hardness Score; FBS, fasting blood sugar; FSH, follicle‐stimulating hormone; HbA1c, hemoglobin A1c; HDL, high‐density lipoprotein; IPSS, International Prostate Symptom Score; LDL, low‐density lipoprotein; LH, luteinizing hormone; SHIM, Sexual Health Inventory for Men; TP, total protein.

**TABLE 2:** Symptoms of sexual dysfunction.

Sexual dysfunction	139 (19.3%)
Erectile dysfunctoin	88 (12.2%)
Ejaclatory dysfunction	66 (9.1%)
Decreased libido	35 (4.9%)

**TABLE 3:** Clinical characteristics of the SD and SN groups.

	SD	SN	*p*
N (%)	139 (19.3)	580 (80.7)	
Age (years)	38.4 ± 7.6	34.8 ± 6.1	< 0.01
BMI (kg/m^2^)	23.2 ± 2.8	22.7 ± 3.0	NS
Smoking (%)	21 (15.1)	92 (15.9)	NS
Rt. testicular volume (mL)	18.9 ± 4.3	19.8 ± 4.2	< 0.05
Lt. testicular volume (mL)	20.1 ± 4.4	20.8 ± 3.8	< 0.05
IPSS	4.4 ± 4.4	3.2 ± 3.5	< 0.01
BDI	6.8 ± 6.5	5.0 ± 5.0	< 0.01
SHIM	13.4 ± 5.6	20.2 ± 4.2	< 0.01
EHS	2.9 ± 0.9	3.6 ± 0.6	< 0.01
TP (g/dL)	7.4 ± 0.4	7.4 ± 0.3	NS
Alb (g/dL)	4.7 ± 0.3	4.8 ± 0.3	< 0.01
AST (U/L)	25.2 ± 20.0	22.3 ± 10.3	< 0.01
ALT (U/L)	29.2 ± 21.9	26.2 ± 23.9	< 0.01
γ‐GTP (U/I)	42.3 ± 41.6	35.2 ± 34.3	< 0.01
BUN (mg/dL)	13.10 ± 2.85	12.97 ± 2.85	NS
Creatinine (mg/dL)	0.835 ± 0.117	0.831 ± 0.102	NS
T‐cholesterol (mg/dL)	197.7 ± 32.7	195.9 ± 33.1	NS
Triglyceride (mg/dL)	100.4 ± 54.8	93.9 ± 71.3	< 0.05
LDL‐cholesterol (mg/dL)	120.8 ± 30.9	119.8 ± 30.6	NS
HDL‐cholesterol (mg/dL)	60.0 ± 13.9	60.1 ± 13.2	NS
FBS (mg/dL)	89.4 ± 11.6	86.6 ± 8.7	< 0.05
HbA1c (%)	5.3 ± 0.3	5.3 ± 0.3	NS
LH (IU/L)	3.3 ± 2.0	3.4 ± 1.8	NS
FSH (IU/L)	4.7 ± 5.7	4.2 ± 3.1	NS
Total testosterone (ng/mL)	5.2 ± 1.8	5.5 ± 1.9	NS
Semen volume (mL)	3.0 ± 1.5	3.4 ± 1.6	< 0.05
Sperm count (×10^6^/mL)	75.2 ± 46.7	70.7 ± 41.9	NS
Sperm motility (%)	59.8 ± 17.2	62.6 ± 17.0	NS

*Note:* All measurements are presented as mean ± standard deviation.

Abbreviations: γ‐GTP, gamma‐glutamyl transpeptidase; ALB, albumin; ALT, alanine aminotransferase; AST, aspartate aminotransferase; BDI, Beck Depression Inventory; BMI, body mass index; BUN, blood urea nitrogen; EHS, Erection Hardness Score; FBS, fasting blood sugar; FSH, follicle‐stimulating hormone; HbA1c, hemoglobin A1c; HDL, high‐density lipoprotein; IPSS, International Prostate Symptom Score; LDL, low‐density lipoprotein; LH, luteinizing hormone; SD, sexual dysfunction group; SHIM, Sexual Health Inventory for Men, SN, normal sexual function group; TP, total protein.

**TABLE 4:** Factors associated with the presence of sexual dysfunction: univariate and multivariate logistic regression analyses.

Variable	Category	*N*	Sexual dysfunction *N* (%)	Univariate models			Multivariate models (Backward elimination method: *p* < 0.05)		
				Odds		[*p*]	Odds		[*p*]
				Point estimate	95% CI	Category *p* value	Point estimate	95% CI	Category *p* value
Age (years)	21 to < 30	103	8 (7.8)	Reference	—	[< 0.001 ***]	Reference	—	[< 0.001***]
	30 to < 34	143	16 (11.2)	1.496	(0.615, 3.639)	0.375	1.401	(0.565, 3.471)	0.466
	34 to < 39	157	38 (24.2)	3.791	(1.689, 8.510)	0.001 **	3.749	(1.641, 8.568)	0.002**
	39 to 63	144	37 (25.7)	4.105	(1.822, 9.251)	< 0.001 ***	3.718	(1.623, 8.519)	0.002**
BMI (kg/m^2^)	15.7 to < 20.8	132	16 (12.1)	0.933	(0.450, 1.936)	0.852	0.820	(0.383, 1.752)	0.608
	20.8 to < 22.5	132	17 (12.9)	Reference	—	[0.012 *]	Reference	—	[0.022*]
	22.5 to < 24.2	138	30 (21.7)	1.879	(0.981, 3.601)	0.057	1.924	(0.980, 3.780)	0.057
	24.2 to 36.3	145	36 (24.8)	2.234	(1.186, 4.210)	0.013*	1.913	(0.986, 3.713)	0.055
History of smoking	No	454	83 (18.3)	Reference	—	—			
	Yes	93	16 (17.2)	0.929	(0.516, 1.673)	0.806			
Testicular volume (minimam; mL)	2 to < 18	131	32 (24.4)	1.863	(1.086, 3.197)	0.024*			
	18 to < 20	81	13 (16.0)	1.102	(0.549, 2.211)	0.784			
	20 to < 22	105	20 (19.0)	1.356	(0.738, 2.492)	0.326			
	22 to 30	230	34 (14.8)	Reference	—	[0.141]			
IPSS	0 to < 1	83	12 (14.5)	Reference	—	[0.017*]	Reference	—	[0.199]
	1 to < 2	123	13 (10.6)	0.699	(0.302, 1.619)	0.404	0.742	(0.290, 1.900)	0.534
	2 to < 5	194	37 (19.1)	1.394	(0.686, 2.833)	0.358	1.273	(0.566, 2.862)	0.560
	5 to 19	147	37 (25.2)	1.990	(0.972, 4.073)	0.060	1.693	(0.726, 3.950)	0.223
BDI	0 to < 2	136	15 (11.0)	Reference	—	[0.028 *]	Reference	—	[0.021*]
	2 to < 4	127	21 (16.5)	1.598	(0.784, 3.257)	0.197	1.634	(0.781, 3.418)	0.192
	4 to < 7	130	25 (19.2)	1.921	(0.962, 3.835)	0.064	1.768	(0.864, 3.618)	0.119
	7 to 42	154	38 (24.7)	2.643	(1.380, 5.060)	0.003**	2.853	(1.448, 5.619)	0.002**
Total protein (g/dL)	6.1 to < 7.2	130	28 (21.5)	1.430	(0.781, 2.617)	0.247			
	7.2 to < 7.4	96	13 (13.5)	0.816	(0.393, 1.692)	0.584			
	7.4 to < 7.7	172	34 (19.8)	1.283	(0.721, 2.282)	0.396			
	7.7 to 8.5	149	24 (16.1)	Reference	—	[0.381]			
ALT (U/L)	7 to < 15	120	13 (10.8%)	Reference	—	[0.014*]	Reference	—	[0.260]
	15 to < 20	147	21 (14.3%)	1.372	(0.656, 2.870)	0.401	1.024	(0.423, 2.478)	0.958
	20 to < 30	143	35 (24.5%)	2.667	(1.337, 5.320)	0.005**	1.681	(0.636, 4.443)	0.295
	30 to 307	137	30 (21.9%)	2.308	(1.142, 4.665)	0.020*	0.858	(0.270, 2.729)	0.795
Creatinine (mg/dL)	0.58 to < 0.76	121	26 (21.5%)	Reference	—	[0.071]			
	0.76 to < 0.83	142	22 (15.5%)	0.670	(0.357, 1.256)	0.211			
	0.83 to < 0.89	126	15 (11.9%)	0.494	(0.247, 0.986)	0.046 *			
	0.89 to 1.17	158	36 (22.8%)	1.078	(0.609, 1.909)	0.796			
Triglyceride (mg/dL)	19 to < 54	134	19 (14.2)	1.157	(0.573, 2.335)	0.685	1.079	(0.497, 2.343)	0.848
	54 to < 74	136	17 (12.5)	Reference	—	[0.018*]	Reference	—	[0.717]
	74 to < 111	139	27 (19.4)	1.687	(0.873, 3.263)	0.120	1.099	(0.519, 2.329)	0.805
	111 to 658	138	36 (26.1)	2.471	(1.310, 4.660)	0.005**	1.490	(0.700, 3.171)	0.301
HbA1c (%)	4.6 to < 5.1	99	15 (15.2)	Reference	—	[0.497]			
	5.1 to < 5.3	141	22 (15.6)	1.035	(0.507, 2.113)	0.924			
	5.3 to < 5.5	164	35 (21.3)	1.519	(0.782, 2.952)	0.217			
	5.5 to 6.8	143	27 (18.9)	1.303	(0.653, 2.601)	0.452			
Testosterone (ng/mL)	1.15 to < 4.22	136	30 (22.1)	1.356	(0.746, 2.467)	0.318			
	4.22 to < 5.34	135	25 (18.5)	1.089	(0.587, 2.021)	0.787			
	5.34 to < 6.63	139	24 (17.3)	Reference	—	[0.452]			
	6.63 to 14.70	137	20 (14.6)	0.819	(0.429, 1.564)	0.546			

Abbreviations: ALT, alanine aminotransferase; BDI, Beck Depression Inventory; BMI, body mass index; HbA1c, hemoglobin A1c; IPSS, International Prostate Symptom Score.

*p<0.05, **p<0.01, ***p<0.001.

## 4 Discussion

The subjects of this study were newlywed men or men just before marriage and were currently hoping to have children. The comparison between the SD and SN groups showed testicular volume was slightly larger on both sides in the SN group. Regarding semen parameters, the SN group also had significantly greater semen volume. However, the influence of age on these findings cannot be ruled out [8, 9]. Based on common sense, one might assume these men constitute the most sexually active group. However, the results of this study reveal a surprising fact: even among men in this generation who strongly desire children, approximately one in five (19.3%) report already experiencing sexual dysfunction. Looking at the breakdown of sexual dysfunction, a significant percentage (12.2%) of men reported experiencing ED. Simultaneously, it is surprising that 9.1% of men complained of ejaculatory disorders, which have not been considered clinically significant until now. This ejaculation disorder likely includes both premature ejaculation and delayed ejaculation. The most surprising finding is that 4.9% of men already experienced reduced libido despite desiring to have children. These results may reflect Japan's current situation, where the number of births declines annually and the low birthrate continues to worsen. The rate of sexual dysfunction as a cause of male infertility is increasing, and the 2023 national survey on male sexual dysfunction conducted by JSSM reported high rates of ED and decreased libido among young men. In fact, the prevalence of ED in males aged 20–24, 25–29, and 30–34 years was 26.6%, 22.0%, and 16.4%, respectively. Furthermore, the rate of those who reported having no or little sexual desire in males aged 20–24, 25–29, and 30–34 years was 21.2%, 14.7%, and 15.8%, respectively [7]. These findings are relatively comparable to those of the present study. Although the reason for the low sexual activity is not clearly understood, it has been speculated that, even among younger generations, frequent viewing of pornographic videos may reduce sexual desire [10]. Furthermore, studies indicate that higher rates of pornographic video consumption correlate with an increased prevalence of ED [11]. Considering these findings, one concern is that even men hoping to have children may face issues in today's environment, where the internet and social media make pornographic videos easily accessible. In other words, it is plausible that developing a habit of routinely exposing oneself to excessive sexual stimuli could gradually diminish sexual activity.

Univariate analyses identified age, obesity (i.e., BMI), LUTS (i.e., IPSS), depressive symptoms (i.e., BDI), and elevated ALT and triglycerides as factors associated with sexual dysfunction. Numerous reports have documented the close relationship between LUTS and sexual dysfunction [12, 13, 14]. Even in young men, factors such as stress, anxiety, sleep deprivation, and excessive caffeine intake can lead to sympathetic nervous system dominance, causing stress‐related autonomic symptoms [15, 16]. This can result in the simultaneous occurrence of bladder neck/prostate smooth muscle contraction and impaired relaxation of the penile cavernosal smooth muscle. Other hypothesized mechanisms include reduced nitric oxide synthase activity, chronic inflammation and oxidative stress, and pelvic floor muscle dysfunction. Regarding liver function, ALT is an aminotransferase between alanine α‐ketoglutarate, glutamic acid, and pyruvic acid, and is highly specific for hepatocytes. Tissue damage releases the enzyme into the blood and increases its activity. Numerous studies have linked liver diseases such as cirrhosis, alcoholic liver disease, and hepatitis with ED [17, 18, 19]. We recently reported that an elevated FIB‐4 index (an indicator of liver fibrosis) is associated with sexual dysfunction [20]. In this context, the finding of elevated ALT is noteworthy. Since triglycerides promote atherosclerosis, it is reasonable to infer that they may impair erectile function. The remaining factors such as age, obesity, and depressive symptoms were also identified as significant variables in the multivariate analysis, making them the most critical outcomes for men who are considering initiating fertility treatment.

Regarding age, the 2023 JSSM national survey also demonstrated that the prevalence of ED generally increases with age [7]. It is well established that aging is a risk factor for ED, as outlined in the *JSSM Guidelines for the Management of Male Sexual Dysfunction*, as well as in the guidelines of the AUA [21]. Various reports have also been published on the relationship between obesity and sexual function [22]. It has been reported that an increase in BMI leads to a decline in sexual function [23, 24]. Moreover, obesity readily induces metabolic factors, and consequently, the mechanism by which it causes ED via atherosclerosis is easily inferred [25, 26]. Furthermore, obesity is strongly associated with hypogonadism [27, 28]. Although serum testosterone levels were not a significant factor in this study, the possibility that various endocrine factors are involved cannot be ruled out. The findings from this study, indicating that worsening depressive symptoms, as assessed by the BDI, correlate with a higher rate of sexual dysfunction, suggest that mental health factors exert an extremely strong influence on the sexual function of men beginning fertility treatment [29, 30]. A Japanese study using symptom‐specific questionnaires for sexual function and depression in 1419 subjects reported an odds ratio of 2.02 for ED in the presence of depressive symptoms [31]. When facing work‐related issues, caring for elderly parents, or experiencing financial anxiety, sexual desire may diminish. Furthermore, the pressure to engage in intercourse precisely on the wife's ovulation day for fertility purposes and the expectation to achieve vaginal ejaculation can further destabilize an individual's mental state [32, 33].

This study had several limitations. First, blood tests and semen tests were performed only once. As this is a chart‐based retrospective study, and premarital bridal checkups are typically requested only once, it was not possible to collect data from multiple visits. Second, the definitions for ED, ejaculatory dysfunction, and decreased libido were not clearly specified. They were all based on patient complaints. Third, information on medication and past history for other diseases was not complete because this was a chart‐based retrospective study. Despite these limitations, this study found that approximately one in five (19.3%) men who have started trying to conceive exhibit sexual dysfunction, with psychogenic factors playing a significant role due to the substantial impact of mental health. This aligns with our clinical experience and we are confident it represents valuable evidence.

## 5 Conclusion

A notable new finding is that sexual dysfunction is already observed in approximately one in five Japanese newlywed men or men just before marriage when beginning attempts to conceive. It is particularly noteworthy that 4.9% of men in this age group, who might otherwise be expected to have peak sexual activity, already exhibit reduced libido. Contributing factors to this decline in sexual function include depression and anxiety stemming from the pressure to perform without failure, in addition to aging and obesity. Medical support, including care for these groups, will likely become increasingly necessary.

## Ethics Statement

This study was approved by the institutional review board of Juntendo University Urayasu Hospital (approval no. 2018‐033).

## Conflicts of Interest

The authors declare no conflicts of interest.

## Data Availability

The data that support the findings of this study are available from the corresponding author upon reasonable request.
